# Scale invariance in a nonvibrating magnetic granular system

**DOI:** 10.1038/s41598-020-68345-z

**Published:** 2020-07-10

**Authors:** G. Torres-Vargas, R. Fossion, F. Donado, F. López-González, C. Tapia-Ignacio

**Affiliations:** 1 Instituto de Ciencias Básicas e Ingeniería, Universidad Autónoma del Estado de Hidalgo, 42184 Hidalgo, Mexico; 2 Posgrado en Ciencias Naturales e Ingeniería, Universidad Autónoma Metropolitana Cuajimalpa, 05348 Mexico, Mexico; 3 Instituto de Ciencias Nucleares, Universidad Nacional Autónoma de México, 04510 Mexico, Mexico; 4 Centro de Ciencias de la Complejidad (C3), Universidad Nacional Autónoma de México, 04510 Mexico, Mexico

**Keywords:** Statistical physics, Nonlinear phenomena

## Abstract

A nonvibrating magnetic granular system is studied by using a time series approach. The system consists of steel balls confined inside a circular wall that surrounds a glass plate. Kinetic energy is provided to the particles by the application of an external vertical time-dependent magnetic field of different amplitudes. We carried out a characterization of the system dynamics through the measurement of the correlations present in the time series of positions, in the *x*-direction, of each particle. In particular, by performing Fourier spectral analysis, we find that the time series are fractal and scale invariant, in such a way that the corresponding Fourier power spectra follow a power law $P(f)\propto 1/f^\beta$, with $0<\beta <2.5$. More specifically, we find that the values of $\beta$, and therefore the strength of the correlations, increase as the magnetic field also increases. In this way, the present system constitutes an experimental model to generate correlated random walks. Additionally, we show how the introduction of a constant magnetic field breaks down this scale invariance property in the positions of each particle. Finally, we confirm the above results by applying detrended fluctuation analysis.

## Introduction

Colloidal and granular systems have been used to model successfully glass-forming liquids^1–6^. Granular systems are composed of relatively large objects that can be observed macroscopically^7^. Usually, a granular system is vibrated vertically to provide energy to the particles^8–13^, although several mechanisms have been used to fluidize particle motions^14,15^. One of the advantages of such systems, for instance, is that the interparticle interactions can be modulated by active controls^15–18^. Another advantage is that their dynamics and structural properties can be studied directly from the positions of the particles, in a straightforward way. The above is because their dynamics is comparatively slow and can be tracked using standard methods.

In previous work, we have studied a 2*D* dimensional nonvibrating granular system composed of steel balls, with a permanent dipole moment, $\mu$, moving on a flat area limited by a circular wall. The particles are provided with kinetic energy through the application of a time-dependent external magnetic field $B_o$^16,19,20^. The kinetic energy of the particles is rapidly lost, but it is quickly compensated by the oscillating magnetic field, which continuously adds energy preventing them from becoming immobile. Although the system is not in thermodynamic equilibrium, it can reach a stationary state. The system behavior meets the criteria to be considered as an Ornstein–Uhlenbeck stochastic process, and therefore, its behavior is similar to a system in thermal equilibrium^18^. In our case, the amplitude of the oscillating magnetic field, $B_o$, is proportional to the effective granular temperature, $T_E$^16^.

In particular, we carried out quenching experiments, from an initial state with the same initial temperature to a final state with lower temperature, for a constant particle concentration. This protocol to cool the system has also been used in Refs.^19,20^. We first consider the case where the particles in the system are only under the effect of a vertical sinusoidal magnetic field with different amplitudes. Secondly, a constant magnetic field $B_c$ is added. This last situation was already studied by means of the calculation of the mean-square displacement, radial distribution function, intermediate scattering function, Maxwell-Boltzmann distribution and effective potentials^16,19,20^.

In the present contribution, we introduce standard techniques from time series and signal analysis, namely, Fourier analysis (FA)^21–24^, and detrended fluctuation analysis (DFA)^25–28^. These mathematical tools have been successfully applied in the study of diverse complex phenomena, where often also scale invariant and fractal dynamics including long-range correlations have been observed, e.g., finance^29^, ecology^30^, climate^31^, circadian cycles^32^, heart rate^33^, electroencephalograms^34^, music^35^, random-matrix eigenspectra^36^ and quasi-bidimensional turbulent flows^37^. Here, we calculate the Fourier power spectrum, *P*(*f*), from the time series of positions, in the *x*-direction, of each particle in the aforementioned experimental model, where *f* is the frequency of the periodic modes in which the time series is decomposed. In this way, a set of power spectra is obtained from the entire system for each value of $B_o$. From all these spectra, an average power spectrum is calculated, which follows a power law,

1 $$\begin{aligned} P(f)\propto 1/f^\beta , \end{aligned}$$

indicating that the time series are scale invariant (fractal). The exponent $\beta$ is a measure of the strength for the long-range correlations present in the time series^38^. We found that the values of $\beta$ increase as the value of $B_o$ also increases, such that $0<\beta <2.5$. Thereby, a characterization of the average dynamics of all particles in the system, based on the scale invariance properties, is achieved in a straightforward way. In other words, we characterize the system dynamics through the quantification of the correlations present in the time series of particle positions. In order to avoid a wrong detection of apparent long-range correlations, we also analyze the fractal properties of the time series by applying DFA, to confirm the results found by applying FA.

In this manner, the present research also seeks to contribute to the field of time series analysis through the presentation of a particular mechanism to generate $1/f^\beta$ noise in an experimental way. When in a complex system, its multiple components interact locally, somehow a new global behavior emerges at the system level that cannot be predicted ab initio from the local interactions. Often, this global behavior of complex systems is characterized by $1/f^\beta$ noise and multiple generating mechanisms have been proposed to model these fractal time series. Examples of such mechanisms include intermittency^39^, electronic circuits^40^, self-organized criticality^41^, lognormal distributions^42^, multiscaled randomness^43^, Fourier filters^38^, random matrix theory^36^, memoryless nonlinear transformations^44^, and anomalous diffusion in complex media^45^. Most of the proposed mechanisms are based on theoretical models, whereas the system which we studied here offers the possibility to experimentally generate $1/f^\beta$ time series that can smoothly transition over a wide range of $\beta$ scaling exponent values.

The paper is organized as follows. First, we explain how FA and DFA are able to characterize the long-range correlations present in the time series of particle positions. We discuss the results of applying FA and DFA to the time series, and compare both approximations. Additionally, we show how the power law behavior breaks down when a constant magnetic field is applied. Then we present our conclusions. Finally, we describe the experimental setup, and describe in a general way how is the dynamics of the particles in the system.

## Results and discussion

### Time series analysis

In the following we describe the techniques, from time series and signal analysis, employed to quantifying long-range correlations in time series, namely, FA and DFA. In this way, a characterization of the average dynamics of all particles in the system, based on the scale invariant properties, is achieved. As we will see, the nonvibrating magnetic granular system studied here, adds up to many other systems where $1/f^\beta$ noise appears^31^. However, we find that scale invariance breaks down in the presence of a constant magnetic field component $B_c$.

**Figure 1:**
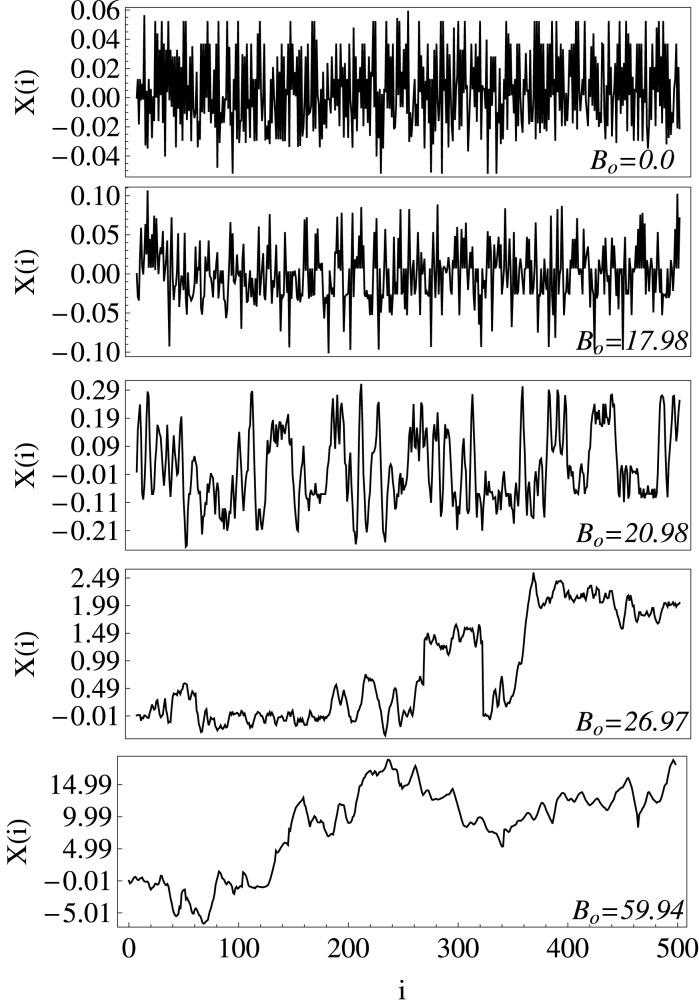
Representative time series of particle positions in the *x*-direction, $X(i)=(x_1,x_2,\ldots ,x_M)$; $i=1,\ldots ,M$, with $M=500$, for several values of the amplitude of the magnetic field $B_o$ (Gauss), and a constant packing fraction $\phi _s = 0.17$. As $B_o$ increases, the times series becomes more correlated. The positions are given in dimensionless units.

Examples of times series of the positions, in the *x*-direction, corresponding to the trajectories of certain particles, are shown in Fig. 1. There, two effects can be appreciated for increasing $B_o$, on the one hand the amplitude of the time series becomes larger, reflecting larger displacements for the particles, and on the other hand the time series becomes less irregular and more persistent, reflecting an increase of the correlations in the positions of the particles.

#### Fourier analysis (FA)

It is well known that the FA is a technique by means of which it is possible to decompose a signal in a combination of sinusoidal waves, each with a certain frequency *f* and amplitude *A*(*f*). The Fourier power spectrum, *P*(*f*), is a representation of such decomposition. In practice, *P*(*f*) can be calculated as the square of the absolute value of the Fourier transform of a time series^21–24^. According to the Wiener-Khinchine theorem, the total power of the power spectrum is equal to the variance of the time series^38^. The correlations present in a time series can be estimated via the Fourier power spectrum in the following way. For a periodic time series, *P*(*f*) has a discrete number of peaks which indicate the dominant frequencies constituting the series, while for a random non-correlated series, *P*(*f*) is continuous and flat because there are no dominant frequencies. The latter is known as *white noise*. The power spectrum of a non-periodic but correlated time series is also continuous, however, due to the correlations, some frequency scales will contribute more to the series than others.

In the case of a *fractal* time series, i.e., a time series whose fragments are statistically self-similar to the entire series, the power spectrum must also be scale invariant and follows a power law $P(f)\propto 1/f^\beta$, where $\beta$, known as the spectral density exponent, is a measure of the strength for the correlations present in the time series^38^. In a log–log representation, the power law is translated to a straight line, being $\beta$ its slope. It is found that $\beta <0$ for anti-correlated time series, $\beta =0$ for neither correlated nor anti-correlated time series, and $\beta >0$ for correlated time series^30,46^. In this context, a time series is called *Brownian noise* if $\beta =2$. We want to emphasize that the time series we are going to study correspond to time series of particle positions, *X*(*i*), and *not displacements*, $\Delta X(i)=X(i)-X(i-1)$, with respect to the previous position $X(i-1)$. In this sense, a time series of position in a Brownian type-motion is a correlated series because of it is the integration of white noise.

#### Detrended fluctuation analysis (DFA)

DFA is an analysis technique to study scaling properties of fractal time series, minimizing the effect of *non-stationary* tendencies^25–28^. DFA avoids the wrong detection of apparent long-range correlations that are actually the result of the non-stationarity of the series. In order to illustrate how the DFA works, let us consider the time series *X*(*i*). First, we integrate *X*(*i*), wherewith we obtain the series *I*(*i*), given by $I(i)=\sum _{j=1}^iX(j)$. After that, *I*(*i*), is divided in windows of length *n*. Next, we adjust a straight line employing a least squares method to the data contained within each window. Such straight line represents the tendency of the data within the windows. We denote the *y* coordinate of the line segments by $y_n(i)$, then we detrend *I*(*i*), subtracting the local tendency, $y_n(i)$ in each window. Then, we estimate the average fluctuation for the window size, *F*(*n*), which is given by $F(n)=\sqrt{\frac{1}{M}\sum _{i=1}^M[I(i)-y_n(i)]^2}$. Finally, we calculate the average fluctuation for a range of time scales (window sizes). A linear relation in a plot of $\log [F(n)]$ versus $\log n$ indicates a scaling of power law (fractal), i.e.,

2 $$\begin{aligned} F(n)\propto n^\alpha . \end{aligned}$$

The slope of such linear relation is equal to the scaling exponent $\alpha$, which characterizes the fluctuations. In particular, we have that $\alpha =0.5$ for white noise, and $\alpha =1.5$ for Brownian noise. The $\alpha$ exponent of DFA is related with the $\beta$ exponent of FA through the relation^47^,

3 $$\begin{aligned} \beta =2\alpha -1. \end{aligned}$$

### Results of time series analysis

**Figure 2:**
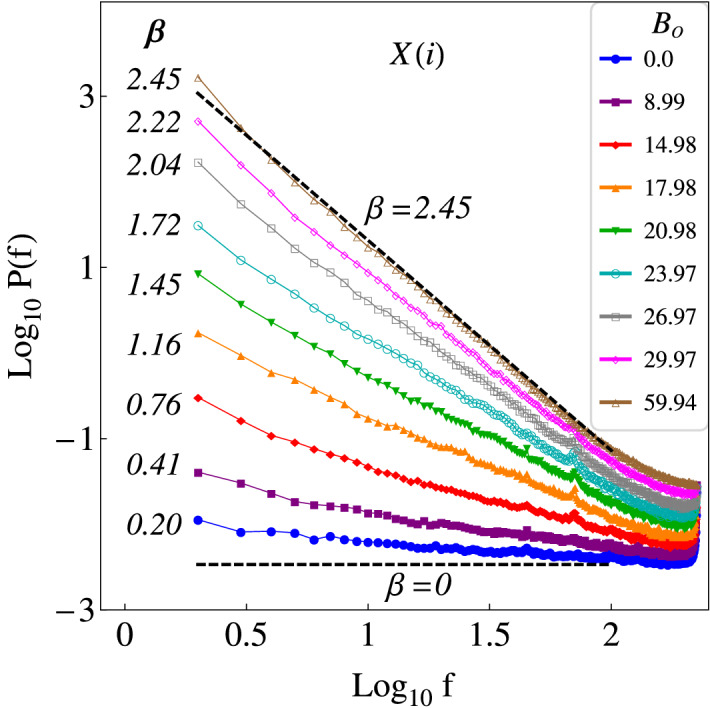
Fourier power spectra of the time series of the particle positions, in the *x*-direction *X*(*i*), for different values of $B_o$, and $\phi _s = 0.17$. The correlation strength $\beta$ is calculated from the slope of a straight line adjusted to each power spectrum in log–log scale. In both cases we can observe that the values of $\beta$ increase, from $\beta \approx 0$ to $\beta \approx 2.5$, as $B_o$ assumes higher values. The results correspond to power spectra averaged over all the particles.

We calculate the Fourier power spectra of the time series of the particle positions in the *x*-direction, $X(i)=(x_1,x_2,\ldots ,x_M)$, being $i=1,\ldots ,M$, with $M=500$, for $\phi _s = 0.17$ and 0 G $\le B_o<63$ G. Since the time resolution is 1/60 s, and each series consist of 500 observations, then the duration of the time series analyzed is approximately of 8.33 s. The corresponding results are shown in Fig. 2. As expected, because of the symmetry of the system, similar results are obtained analyzing the time series of the particle positions in the *y*-direction, *Y*(*i*), which are not presented here. When the intensity of the driving field is very small ($B_o \approx 0$), the field does not have enough power to move the particles, which remain almost immobile. Therefore, the corresponding time series have small variance, and by the Wiener–Khinchine theorem the associated power spectra *P*(*f*) has small total power. Additionally, the power spectrum looks nearly flat ($\beta \approx 0$), indicating that the corresponding time series behave similar to white noise. At somewhat higher energies, the particles begin to move but there are no collisions among them. For higher energies, there are local collisions between particles such that the energy is redistributed. As the energy is increased, the probability of collisions increases as well, and the displacements are such that the power spectra follow a power law with ever increasing slope, and ever increasing total power. In this way, a characterization of the transition in the dynamics exhibited by the particles in the system, as $B_o$ changes, is obtained. The above is achieved in terms of the correlations present in the time series of particle positions, by adjusting a straight line to the power spectra in log–log scale, and then calculating the corresponding value of $\beta$. The transition from uncorrelated to correlated time series is a phenomenon that results from the combination of both individual and collective dynamics of particles.

**Figure 3:**
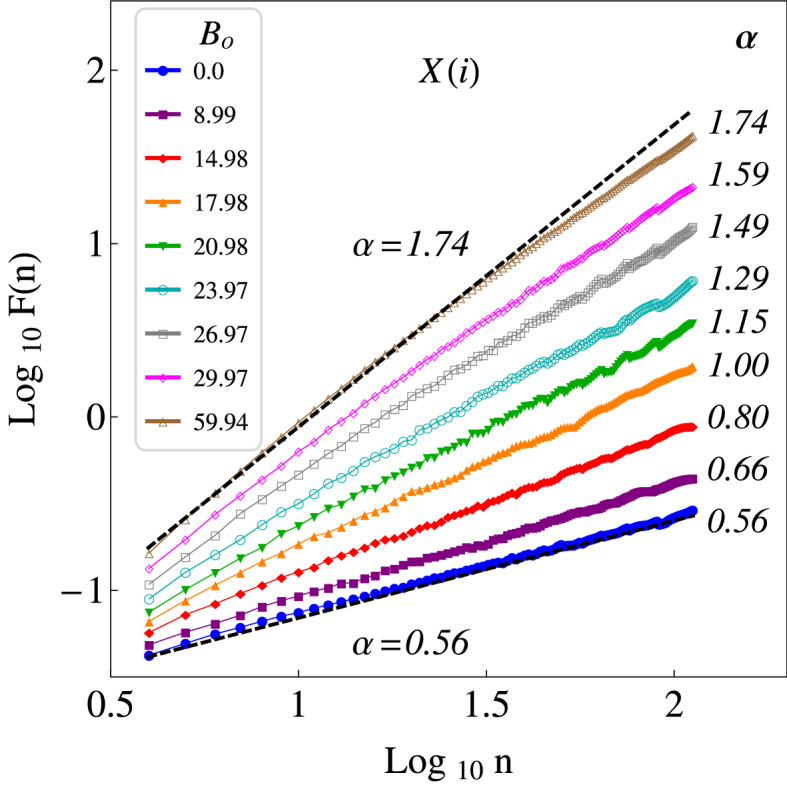
Results of applying DFA to the time series of the particle positions, *X*(*i*), for the same values of $B_o$ and $\phi _s$ in Fig. 2. The exponent $\alpha$ is calculated from the slope of a straight line adjusted to each set of data. In both cases we can observe that the values of $\alpha$ increase, from $\alpha \approx 0.5$ to $\alpha \approx 1.75$, as $B_o$ assumes higher values. The results correspond to an average over all the particles.

**Figure 4:**
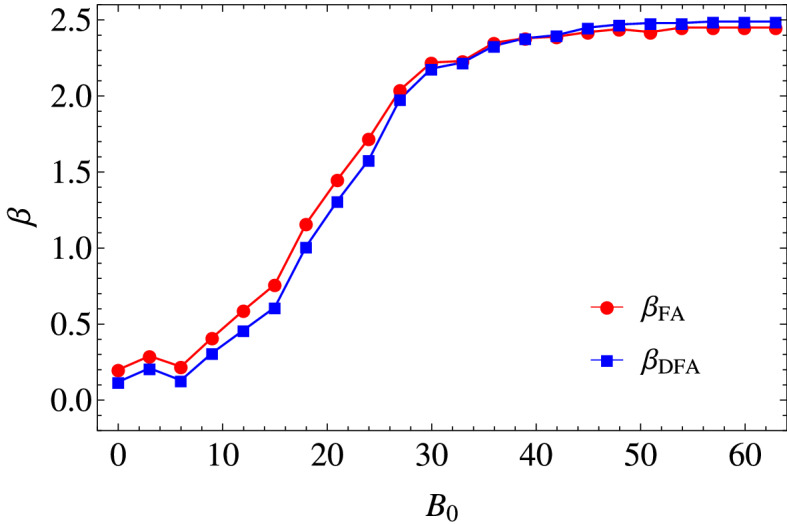
Scaling exponent $\beta$ as function of the amplitude of the magnetic field $B_o$ (Gauss). We calculate the exponents by applying FA ($\beta _{FA}$) and DFA ($\beta _{DFA}$). In both cases, we can observe a transition from $\beta \approx 0$ to $\beta \approx 2.5$, as $B_o$ increases, i.e. a transition from noncorrelated time series to correlated times series. Error bars are not shown since they are of the order of the symbol size.

In Fig. 3, the results are presented of applying DFA to the same time series used for FA. In the present calculations the minimal window size used was $n_{min}=4$, and the maximal window size was $n_{max}=M/4$. A good fit of a straight line can be performed to the data, such that a power law behavior is observed. We can appreciate a transition from $\alpha \approx 0.5$ ($\beta \approx 0$) to $\alpha \approx 1.75$ ($\beta \approx 2.5$), as $B_o$ increases, which is in agreement with the results previously obtained by FA. In Fig. 4, a comparison between the $\beta$-exponents obtained via FA, $\beta _{FA}$, and via DFA, $\beta _{DFA}$, (see Eq. 3) for the times series in the *x*-direction, are presented. In general, we can observe a transition from $\beta \approx 0$ to $\beta \approx 2.5$, as $B_o$ increases, i.e. a transition from noncorrelated time series to correlated times series. For $B_o\gtrapprox 40$ G, the exponent $\beta$ characterizing the power spectrum is essentially unchanged, which indicates that even though $B_o$ is increasing, the correlations in the positions of each particle remain practically unchanged. This, in turn, indicates that there is a limit in $B_o$, such that the correlations stop increasing.

Now, let us consider the effect of an additional constant magnetic field, $B_c$, which we have studied in previous publications^16,19,20^. The term $B_c$ is important because it causes repulsion among the particles, and gives rise to long-range correlations. For example, modulating the intensity of $B_c$ the aggregation of particles can be avoided^15,16^. At this point, it is important to emphasize that the long-range correlations we are studying employing the techniques from signal analysis, FA and DFA, are correlations in the displacements (changes in position in a time step) at different times in a single particle trajectory, and not between trajectories of different particles.

**Figure 5:**
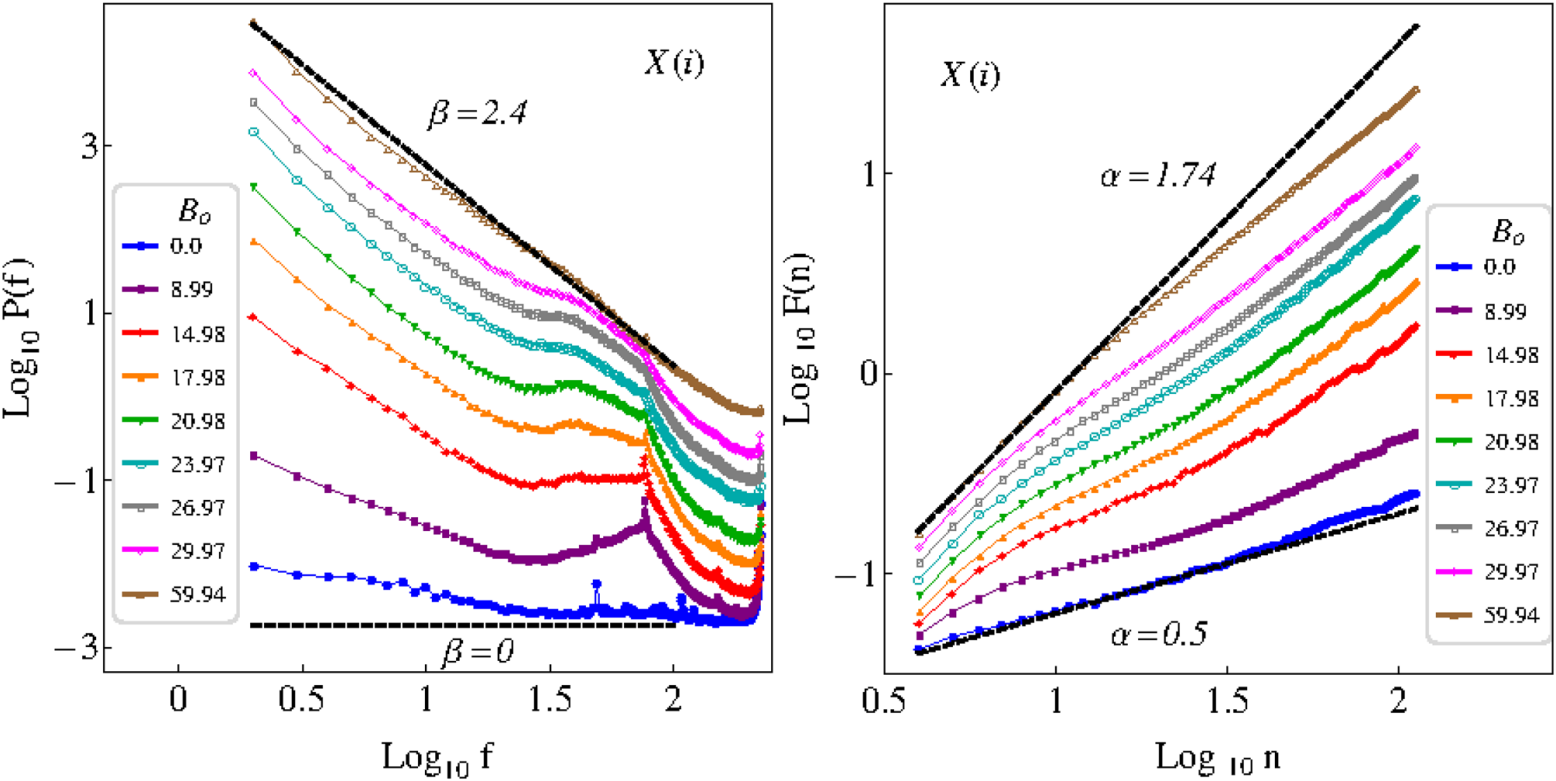
Fourier power spectra of the time series of the particle positions, in the *x*-direction, *X*(*i*) (left panel), and the corresponding results of applying the DFA method to the same time series (right panel), when a constant magnetic field, $B_c = 36$ G, is introduced. The values of $B_o$ and $\phi _s$ considered are the same as in Figs. 2 and 3. Unlike when $B_c = 0$, the power law behavior breaks down. The results correspond to averages over all the particles.

In Fig. 5 are presented the corresponding results obtained after applying FA and DFA to the time series of the particle positions in the *x*-direction, $X(i)=(x_1,x_2,\ldots ,x_M)$, with $M=500$, for $\phi _s = 0.17$, and 0 G $\le B_o<63$ G, as before, but now with an additional nonzero constant magnetic field, $B_c= 36$ G. Unlike when $B_c=0$, scale invariance is lost, and instead of a power law behavior in the power spectra (left panel), now there are dominant broad peaks for low values of $B_o$, which disappear as $B_o$ increases. These peaks occur at the frequency $\nu =9.25$ Hz, which in units of the power spectra (number of oscillations throughout the duration of the time series) occur at (9.25 Hz)$\times (8.33$ s) $\approx 77.073$, such that $\log _{10}(77.073)\approx 1.887$. Dominant peaks in a power spectrum are readily interpretable and indicate deterministic (quasi-)periodic behavior, in this case, a local vibration in response to the driving oscillating force of the magnetic field in the presence of a static component $B_c$ which enhances the collective behavior. As the sinusoidal component $B_o$ increases its value, the peaks are broadened by the local collisions among particles such that energy is redistributed. Therefore, the linear fit needs to be performed to the straight part of the power spectrum excluding the broad dominant peak corresponding to the periodic component.

In Fig. 5 (right panel) we can appreciate that the results of DFA also exhibits a breakdown of the scale invariance. Here, the behavior is more complex, with two scaling regimes and a broad crossover region in between, which makes a fitting procedure difficult. Such crossover behavior for DFA analysis has been found for, e.g., finance^48^, EEG^49^, heart rate^50^ and electroseismicity^51^, which has been attributed to the presence of periodic components in the time series. The above raises important methodological questions regarding the application of DFA to time series which contain also periodic components apart from fractal behavior^52^. Given that the method of mean squared displacement also presupposes only fractal behavior^53^, the present results indicate that mean square displacement may not be adequate in the presence of periodic components, as is the case here for non vibrating granular models with an added constant magnetic force; we plan to study this more into detail in an upcoming publication.

## Conclusions and remarks

We have introduced two mathematical tools from time series and signal analysis, Fourier analysis (FA) and detrended fluctuation analysis (DFA), to perform a characterization of the particles dynamics in a non-vibrating magnetic granular system, under the action of a magnetic field, *B*. The above was achieved by the application of FA and DFA to the time series of the particle positions in the *x*-direction. In particular, we considered different values of the amplitude of the magnetic field, $B_o$, and a constant packing fraction, $\phi _s=0.17$. In this way, a quantification of the correlations in the time series was carried out. We obtained that the time series are scale invariant (fractal), and the corresponding Fourier power spectra follow a power law $P(f)=1/f^\beta$. We found a transition in the values of $\beta$, from $\beta \approx 0$ to $\beta \approx 2.5$, as $B_o$ increases, i.e., a transition from non correlated time series to correlated times series. The power law behavior was confirmed by the application of DFA to the same time series of particle positions. For $B_o\gtrapprox 40$ G, the exponent $\beta$ characterizing the power spectrum is essentially unchanged, which indicates that even though $B_o$ is increasing, the correlations in the positions of each particle remain practically unchanged. It was observed that the introduction of a constant magnetic field, $B_c$, causes a breakdown of the scale invariance in the positions of the particles due to the appearance of periodic collective motion of the particles.

It is important to emphasize that the purpose of the present research is twofold. On the one hand, it seeks to contribute to the study of colloidal and granular systems introducing time series analysis techniques. In particular, we found that FA may be more appropriate than traditional methods such as mean-square displacement, in cases where both fractal and periodic behaviors are present. On the other hand, the present research also seeks to contribute to the field of time series analysis through the presentation of an experimental mechanism to generate $1/f^\beta$ noise, which have already been modeled mathematically through different theoretical approaches, for example, through models like the fractional Brownian motion (fBm), and the fractional Gaussian noise (fGn). Of course, this task implies additional studies and further analysis to those presented here.

## Methods

**Figure 6:**
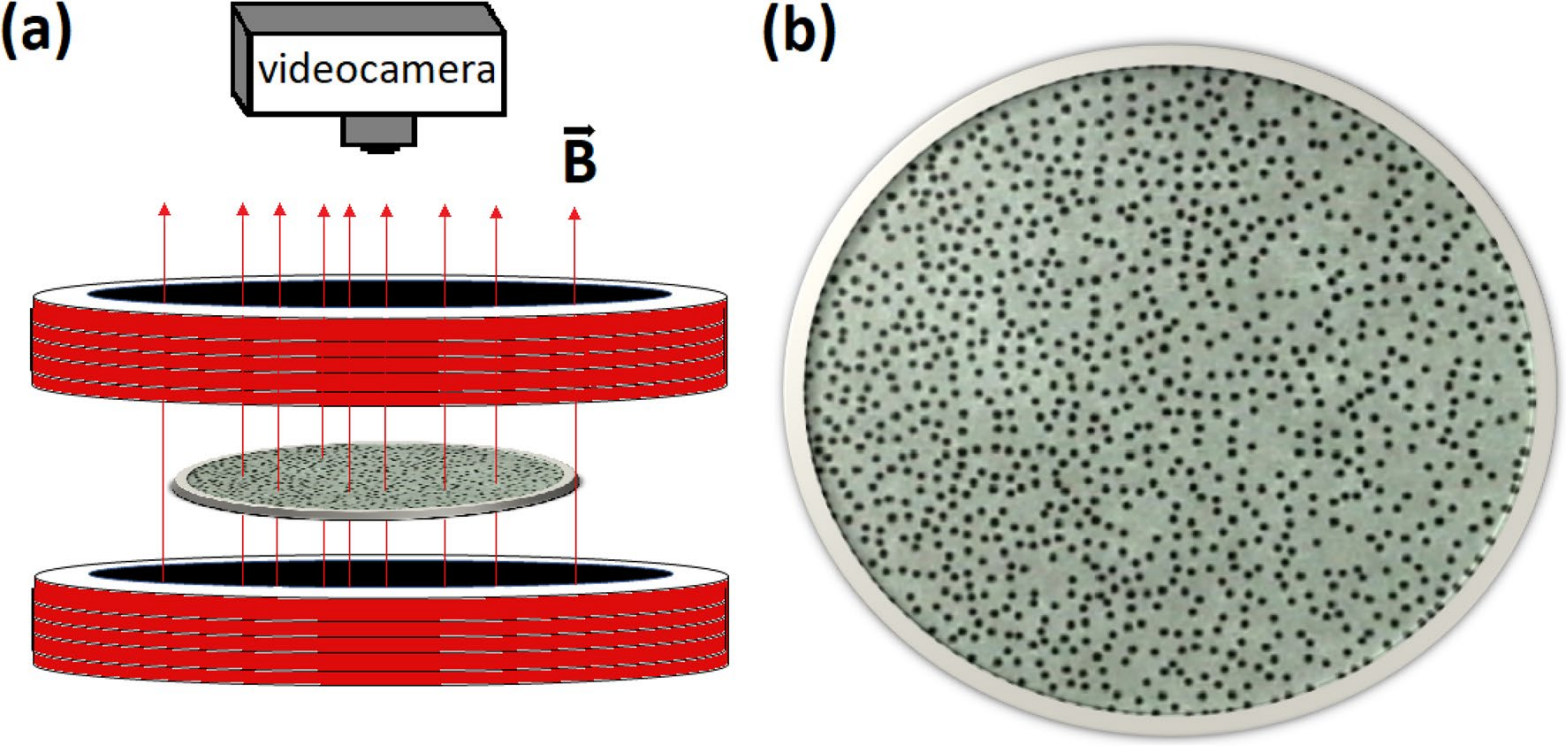
(**a**) Scheme of the experimental setup. The direction of the vertical sinusoidal magnetic field, *B*, is along the *z*-direction, while *x* and *y* are the horizontal directions where the particles move. (**b**) Particles on the glass plate.

We carried out experiments using the experimental setup shown in Fig. 6. The system consists of 1 mm diameter steel balls, confined to move inside a circular wall that surrounds a glass plate, such that the system is open at the top but gravitation maintains the system two-dimensional. We provide kinetic energy to the particles by the application of an external vertical time-dependent magnetic field,

4 $$\begin{aligned} B=B_c + B_o \sin (2\pi \nu t), \end{aligned}$$

which is produced by a pair of Helmholtz coils fed with a power amplifier driven by a function generator. $B_o$ and $\nu$ are the amplitude and the frequency of the unsteady part of the magnetic field, respectively, and $B_c$ is a constant magnetic field contribution. We studied two cases of the constant magnetic field, $B_c=0$ G and $B_c=36$ G, in both cases $\nu =9.25$ Hz, and $B_o$ takes values from 0 to 63 G. The case with $B_c=36$ G was already considered in Refs.^16,19,20^. The two-dimensional packing fraction (particle concentration) is defined by $\phi _{s}=\frac{N \pi \sigma ^{2}}{4A}$, where *N* is the average number of particles in the field of view with area *A*, and $\sigma$ is the diameter of the particle. The particle concentration in these experiments was kept constant at $\phi _s = 0.17$. We record several runs of 2 min of video in a resolution of 411 $\times$ 286 pixels in AVI interlaced format. After carrying out digital processing we obtained a final resolution of 1/60 s. The trajectories of the particles are obtained by using ImageJ and its plug-in Mosaic^54^.

### Granular temperature

The granular temperature can be obtained with the equipartition theorem in a 2D system, which states that $\left\langle \frac{1}{2} m v^{2}\right\rangle =T_E$, where $T_E$ is the effective temperature, and the left term is the average particle kinetic energy. In granular systems, to define an effective temperature the usual practice is to set $k_B = 1$. Such definition in terms of energy is also used in other reports, for instance in Ref.^11^. The effective temperature can also be determined from the Maxwell-Boltzmann velocity distribution. For a wide range of temperatures, we observed that $T_E$ is proportional to $B_o$^16^. In this work we used $B_o$ to characterize the energy of the particles. More specifically, we carry out quenching experiments by means of suddenly decreasing $B_o$, from the same initial temperature to a final state with lower temperature, and analyze 500 frames in a time window of 1.66 s immediately after the system was quenched.

### General dynamics

The maximum energy that a magnetic particle in a sinusoidal magnetic field can receive is $4\mu B_o\nu$^16^. We observed that the energy input is proportional to the frequency and to the amplitude of the unsteady part of the magnetic field. This system is highly dissipative, therefore not all the available energy, $4\mu B_o\nu$, is transformed into particle motion because of the friction of the particles with the plane while rolling, and collisions with other particles. Although energy is quickly lost, it is compensated by the energy input from the magnetic field. This energy input prevents particle motions from stopping.

The relation between energy input and the randomization of particle motion is described elsewhere^16^. Briefly, when a vertical sinusoidal magnetic field is turned on, each particle tends to align with the magnetic field to reach a minimum potential magnetic energy. Starting from a configuration where the particle is aligned in the sinusoidal magnetic-field direction, the field decreases, and eventually it points in the opposite direction. In this new condition, the magnetic energy of the particle is maximum. Thus, the particle rolls to align itself again with the magnetic field. When rolling, the particle moves linearly over the surface. Since a sphere has a neutral balance when it is on a horizontal plane, the particle can roll in any possible direction, which is the origin of the randomization of the motion. Except for low frequencies, changes in particle motion are not synchronous with the magnetic field. Because of rotational inertia, the particle continues rolling in the same direction. For the frequency we used, while a particle is moving, the magnetic field changes direction several times. The resulting motion of the particle is very complex because the torque trying to align it in the field direction changes quickly. Eventually, the particle meets the initial condition described above and it can change its rolling direction to a new unpredictable direction. Of course particle collisions also contribute to increasing changes in particle motions.

**Figure 7:**
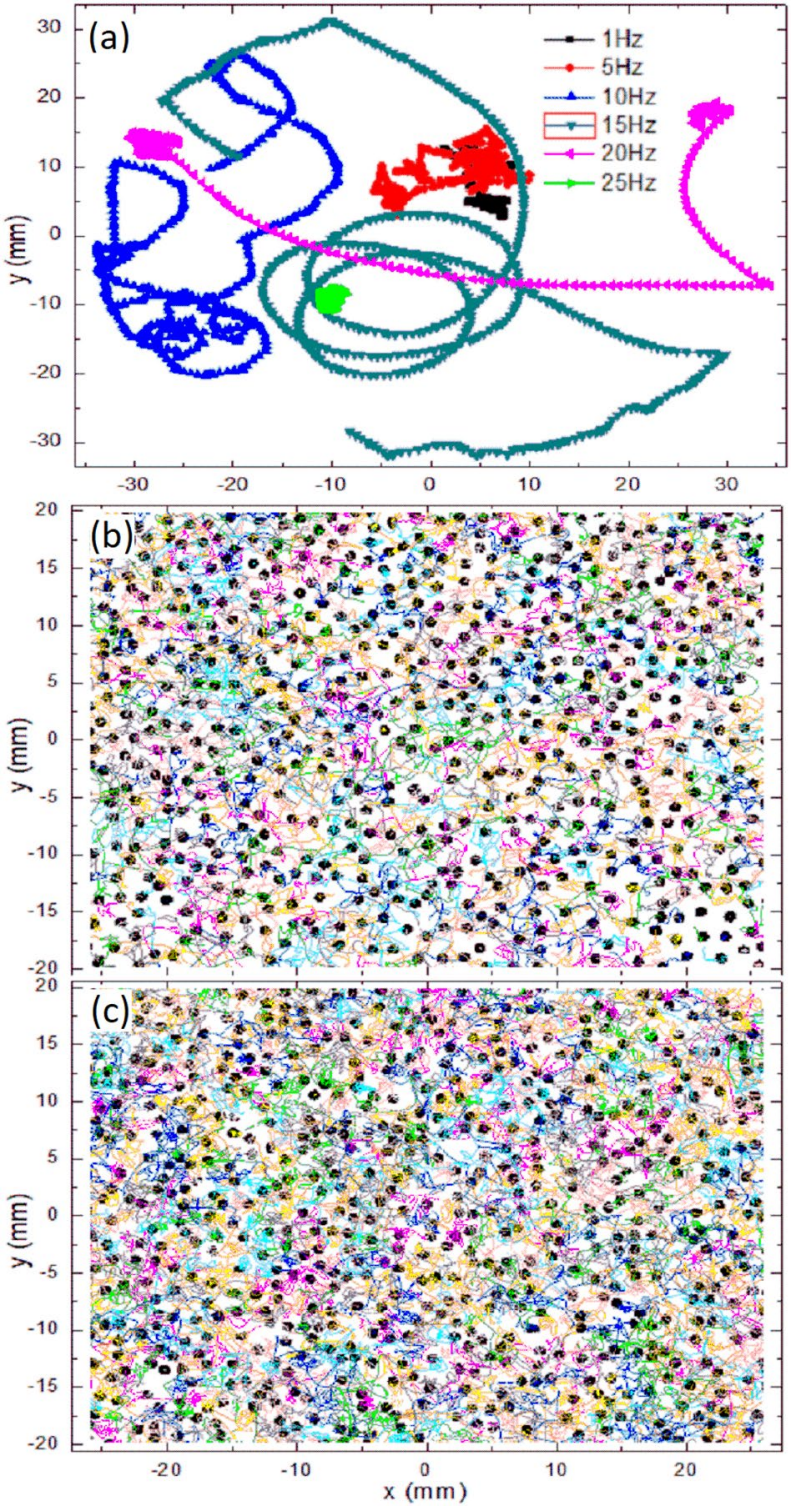
(**a**) Trajectories of a single particle on the glass plate at different frequencies. (**b**) Selected trajectories of a system of particles without constant magnetic field. (**c**) Selected trajectories of a system of particles in the presence of a constant magnetic field. Panel (**a**) shows trajectories for a duration of 8.33 s, whereas in panels (**b**,**c**) the duration is 1.66 s to avoid overlapping trajectories. The frequency in panels (**b**,**c**) is 10 Hz.

The magnetic field is homogeneous in the vertical direction and it does not present a gradient, thus a magnetic dipole is expected to orient itself in the direction of the magnetic field, but not to move in this direction. When an ensemble of particles, each with its own magnetic dipole, interacts, there is a tendency to aggregate head-to-tail to form chains. Below a specific threshold magnetic field intensity, particles remain on the glass surface due to gravity. For stronger magnetic field intensities, we have observed the formation of tetrads, three particles in the base and one particle on the top or even taller structures. Thus, we limited the intensity of the magnetic field in such a way the particles remain in a 2*D* configuration on the glass surface. Figure 7a shows the case of a single particle on the glass plate, and displays several trajectories at different frequencies. It is observed that at the intermediate frequencies of 10 Hz and 15 Hz, the trajectories cover more area than at higher (e.g. 25 Hz) or lower frequencies (e.g., 5 Hz, 1 Hz). The time span is 8.33 s for all trajectories. In the case of a system of particles at low densities, $\phi _s<0.1$, each particle has its own dynamics and the probability that two or more neighboring particles have the same alignment is low, and therefore no repulsive interaction is observed between the corresponding magnetic moments. Thus, apart from sporadic collisions, the motion of each particle is nearly independent from the other particles. In this way, the mechanism of randomization acts at particle level and trajectories continue to behave similar to for the single-particle case of Fig. 7a. For larger particle concentrations, $0.1<\phi _s<0.3$, the probability of collisions increases and consequently the statistics changes. In particular dynamics becomes more restricted than in the cases of lower particle concentrations, as shown in Fig. 7b.

When an additional constant magnetic field $B_c$ is applied, magnetic moments of the individual particles tend to align with this field. For $B_o > B_c$, the amplitude of the magnetic field oscillations increases with the magnitude of $B_o$, such that the effects $B_c$ values are negligible. Particles rolls and move randomly trying to align with the alternating magnetic field. Therefore, as well as in the case of absent of $B_c$, apart from collisions with other particles. The motion of each particle is nearly independent from the other particles and the probability that neighboring particles have the same alignment is low, and then no repulsive interaction is observed between particles. When the value of $B_o$ decreases and approaches $B_c$, the amplitude of magnetic field oscillations decreases and the time in which the magnetic field points in the same direction of $B_c$ increases. In this case, the probability that neighboring particles have the same alignment periodically is increased. Particle motions become more couple and the probability that two particles suffer repulsive interactions is increased. Because of the magnetic field is changing periodically, particle repulsions reach maximums at the same frequency as the oscillating magnetic field. Of course, the dynamics is very complex depending on multiple parameters, such as the particle concentration, the intensity and frequency of the applied magnetic field and the constant magnetic field. When $B_o$ approaches $B_c$, effects of $B_c$ and of crowding are observed and collective effects arise. Figure 7c shows trajectories of particles in a sample when a constant magnetic field is present. The coupling of the particles through the magnetic field is observed by a qualitative reduction of the area covered by the trajectories.
